# Beta oscillations in vision: a (preconscious) neural mechanism for the dorsal visual stream?

**DOI:** 10.3389/fpsyg.2023.1296483

**Published:** 2023-12-13

**Authors:** Giuseppe Di Dona, Luca Ronconi

**Affiliations:** ^1^Division of Neuroscience, IRCCS San Raffaele Scientific Institute, Milan, Italy; ^2^School of Psychology, Vita-Salute San Raffaele University, Milan, Italy

**Keywords:** vision, beta oscillations, magnocellular dorsal stream, visual attention, spatial attention, parietal cortex, perceptual awareness

## Abstract

Neural oscillations in alpha (8–12 Hz) and beta (13–30 Hz) frequency bands are thought to reflect feedback/reentrant loops and large-scale cortical interactions. In the last decades a main effort has been made in linking perception with alpha-band oscillations, with converging evidence showing that alpha oscillations have a key role in the temporal and featural binding of visual input, configuring the alpha rhythm a key determinant of conscious visual experience. Less attention has been historically dedicated to link beta oscillations and visual processing. Nonetheless, increasing studies report that task conditions that require to segregate/integrate stimuli in space, to disentangle local/global shapes, to spatially reorganize visual inputs, and to achieve motion perception or form-motion integration, rely on the activity of beta oscillations, with a main hub in parietal areas. In the present review, we summarize the evidence linking oscillations within the beta band and visual perception. We propose that beta oscillations represent a neural code that supports the functionality of the magnocellular-dorsal (M-D) visual pathway, serving as a fast primary neural code to exert top-down influences on the slower parvocellular-ventral visual pathway activity. Such M-D-related beta activity is proposed to act mainly pre-consciously, providing the spatial coordinates of vision and guiding the conscious extraction of objects identity that are achieved with slower alpha rhythms in ventral areas. Finally, within this new theoretical framework, we discuss the potential role of M-D-related beta oscillations in visuo-spatial attention, oculo-motor behavior and reading (dis)abilities.

## Introduction

Oscillatory activity in the alpha, beta and gamma frequency range (alpha: 8–12 Hz, beta: 15–30 Hz, gamma: 30–80 Hz), have been previously extensively linked to perceptual processes, and also higher-level visual cognition (Tallon-Baudry and Bertrand, 1999; Womelsdorf et al., 2006; Fries, 2009; Engel and Fries, 2010; Martinovic and Busch, 2011; Tan et al., 2013). Their precise role is still matter of intense scientific investigation, but a widely supported idea is that while activity at higher frequency (gamma) would reflect feed-forward stimulus processing restricted to local neural ensembles, activity modulation at lower frequency (alpha and beta) would reflect feedback/reentrant loops and large-scale cortical interactions (Donner and Siegel, 2011; Fries, 2015; Jensen et al., 2015; Sherman et al., 2016; Palmigiano et al., 2017; Palva and Palva, 2018).

Alpha oscillations have been framed as the fundamental rhythm of conscious perception, shaping the internal experience of the sensory world (Valera et al., 1981; VanRullen, 2016; Di Gregorio et al., 2022; Kienitz et al., 2022). Several studies showed that different parameters (e.g., power, phase, frequency) of alpha oscillations determine diverse aspects of visual perception (for recent reviews see: Clayton et al., 2018; Ghiani et al., 2021; Peylo et al., 2021; Keitel et al., 2022; Samaha and Romei, 2023). In line with theoretical proposals that hypothesize a role for alpha oscillations in the temporal sampling of visual information (VanRullen, 2016; Mierau et al., 2017), the speed of alpha oscillations has been found to act as a pacer determining sensory sampling in the brain (Cecere et al., 2015; Samaha and Postle, 2015; Ronconi et al., 2018; Marsicano et al., 2023). Relatedly, the phase of alpha oscillations can determine whether visual signals on a sub-second temporal scale will be temporally integrated or segregated (Wutz et al., 2014; Wutz and Melcher, 2014; Ronconi et al., 2017, 2023; Ronconi and Melcher, 2017). Furthermore, the integrative function of alpha oscillations also operates across features of visual objects. By acting over large scale networks, alpha oscillations would integrate different object features (e.g., orientation, color) which are processed in segregated and specialized brain areas, into one unitary percept (Zhang et al., 2019; Parto Dezfouli et al., 2021).

The role of beta oscillations in perception is much less documented. Nonetheless, mounting evidence in the last decade has linked this oscillatory rhythm to different visual phenomena. In this review, we will try to summarize this emerging literature highlighting the peculiar conditions under which beta oscillations are modulated in visual tasks, its possible functional meaning and its putative neuroanatomical networks. We will propose that beta is likely to be the rhythm of the dorsal (“where”) visual pathway, which would act as a fast track that provides the spatial coordinates for visual perception, acting at a preconscious level to guide the active construction of stimulus identity in the ventral (“what”) stream areas, and plan action (e.g., eye movements) accordingly.

### Spatial integration/segregation and dorsal-to-ventral guidance in perception

The parvocellular-ventral (P-V) and the magnocellular-dorsal (M-D) streams are the two major visual pathways. The P-V stream is characterized by both lower temporal resolution and superior sensitivity to high spatial frequencies, and it is also sensitive to color changes (Kaplan and Shapley, 1986; Kaplan et al., 1990); it is responsible for object identity extraction (Goodale and Milner, 1992). The M-D stream is the other major visual pathway starting from the retina and projecting to occipital and parietal cortices via the M-layer of the lateral geniculate nucleus (Maunsell and Newsome, 1987).

The M-D stream is considered to be color-blind, responds to subtle differences in luminance contrast, and is highly sensitive to low spatial and high temporal frequencies of visual stimulation, thus being the primary pathway for processing motion (Livingstone and Hubel, 1988; Morrone et al., 2000). Moreover, the M-D stream is considered having a central role for contour integration and segregation. There are computational and theoretical reasons indicating that spatial grouping is likely achieved by an interplay between feedforward and feedback activity within the visual system (Roelfsema, 2006; Jehee et al., 2007). While the feedforward connections would promote the representation of visual features within a spatial map (Tootell et al., 1998), feedback activity from higher-level regions would promote the selection of targets according to their spatial location (Foxe and Snyder, 2011). Specifically, fast bottom-up projections to the M-D stream would provide coarse spatial representations facilitating, via recursive feedback from the parietal cortex, the slower and attention-demanding objects identification in ventral stream areas (Vidyasagar, 1999, 2004; Levy et al., 2010; Vidyasagar and Pammer, 2010). Such dorsal-to-ventral communication is thought to promote the activation of receptive fields of appropriate size, resulting in an effective segregation of relevant input (Lamme and Roelfsema, 2000).

Because of the poor spatial resolution of representations carried by the dorsal stream, when encountering spatially complex displays, such as in the case of visually crowded elements, the parietal cortex may erroneously promote binding between targets and irrelevant flankers (Chakravarthi and Pelli, 2011). That would also explain why stimuli that preferentially activate the M-D stream appear to be more vulnerable to visual crowding effect (Atilgan et al., 2020).

When there is the opposing need for spatial integration, such as when solving a contour integration problem, information is bidirectionally exchanged between lateral occipital (i.e., LO1) and parietal regions (i.e., intraparietal sulcus) (Hanslmayr et al., 2013). While LO1 would preferentially respond to orientation (Larsson and Heeger, 2006) and collinearity (Kourtzi et al., 2003) of local elements, synchronization of this region with the parietal cortex would provide a spatial reference by enhancing LO1 neurons firing rates in the relevant locations (Roelfsema, 2006).

### Beta oscillations as the “natural” rhythm of parietal areas

**Figure 1:**
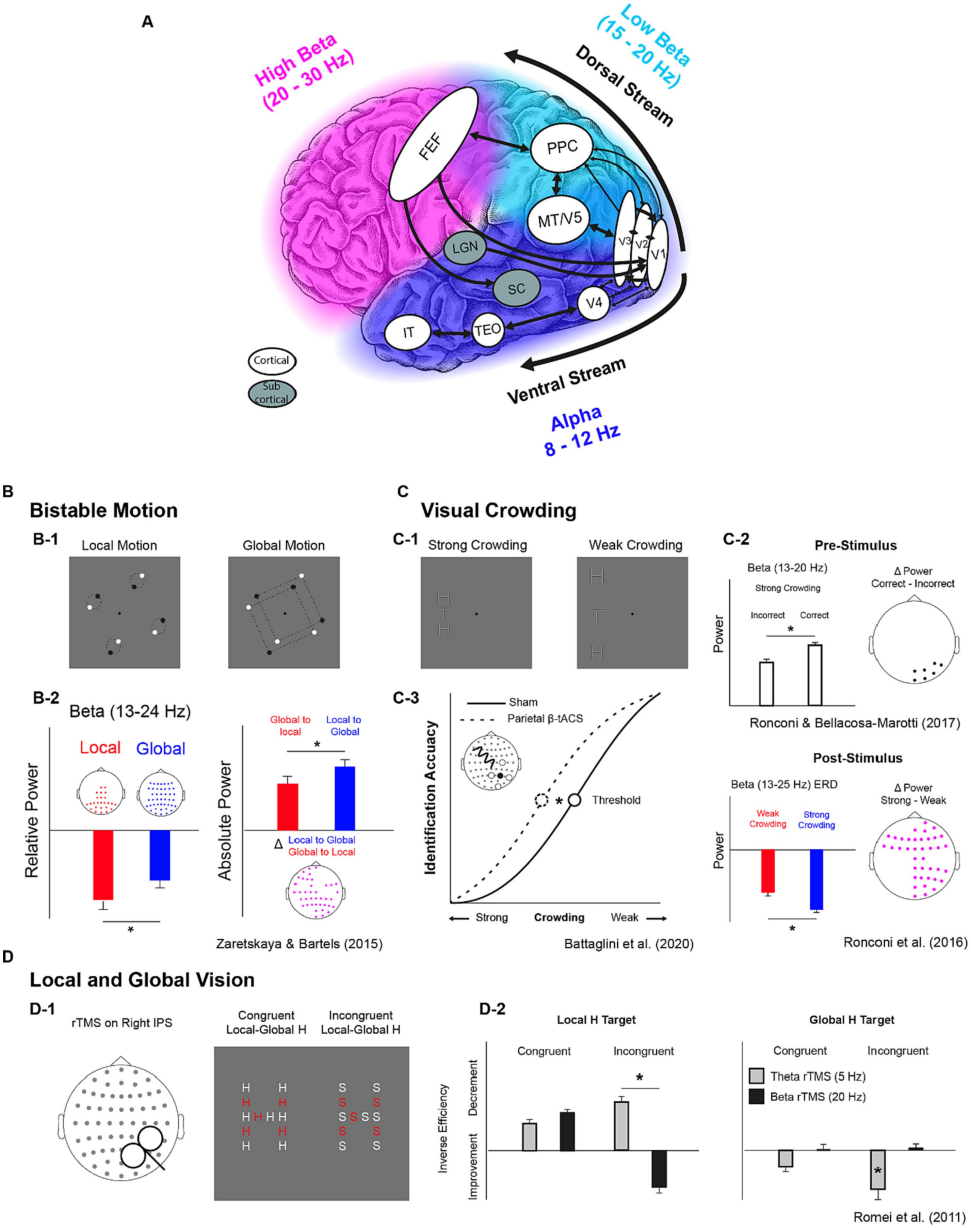
Key structures of visual streams with their natural oscillatory rhythms and their functional properties as highlighted by correlational and causal evidence. **(A)** Key cortical and subcortical structures of the magnocellular dorsal and the parvocellular ventral visual streams. Colors indicate the frequencies of the “natural” oscillatory activity in the 8-30 Hz interval across the occipital and temporal cortex (blue = alpha, 8-12 Hz), the parietal cortex (azure = low beta, 15-20 Hz), and the frontal cortex (fuchsia = high beta, 20-30 Hz) as indicated by Rosanova et al. (2009), Ferrarelli et al. (2012) and Capilla et al. (2022). White balloons indicate cortical structures while gray balloons indicate subcortical ones. Black arrows indicate feedforward/feedback connections. FEF: Frontal Eye FIelds; PPC: Posterior Parietal Cortex; MT/V5: Middle Temporal Cortex/Visual Cortex 5; V1,V2,V3, V4: Visual cortices 1, 2, 3,4; LGN: Lateral Geniculate Nucleus; TEO: Temporo-Occipital Cortex; IT: Inferior Temporal Cortex. **(B-D)** Tasks and results from studies investigating the role of beta oscillations in bistable motion **(B)**, visual crowding **(C)** and local and global Motion **(D)**. Participants were presented with rotating dots and reported when they perceived them as couples of dots rotating along their center or when they perceived two squares enclosed in quadruplets of dots rotating along their center **(B-1)**. A larger decrease of steady-state beta power found for local vs. global perception **(B-2**, leftmost subplot**)**. A larger beta desynchronization was found when switching from local to global with respect to global to local **(B-2**, rightmost subplot**)**. Participants were requested to identify the orientation of a target letter “T” embedded in a vertical array of flanker “Hs” with different levels of visual crowding **(C-1)**. Higher pre-stimulus beta power was associated with correct identification **(C-2**, higher subplot**)** and a larger beta desynchronization was found with strong crowding between target and flankers **(C-2**, lower subplot**)**. In a subsequent study, a higher crowding resilience (lower threshold) was found when participants received beta tACS over right parietal sensors **(C-3)**. Participants performed a Navon task in which they reported the presence of a local stimulus (“H” or “S”) letter in an “H-shaped” or “S-shaped” spatial disposition. Before each trials they either received theta or beta rTMS over the IPS **(D-1)**. In the incongruent condition of the local-target task (“find the local H in the S-shaped disposition”), theta rTMS lead to a decrement in behavioural performance while beta rTMS to an increment, while theta rTMS lead to improvements only in the same condition of the global-target task (“find the global H in an H-like disposition of S letters”) **(D-2)**.

Converging evidence argues for a spatial and functional predominance of beta oscillations in parietal cortices, framing beta as the “natural” rhythm of such networks (Rosanova et al., 2009; Ferrarelli et al., 2012). With a data-driven approach applied to MEG and MRI data from the Open MEG Archive (OMEGA, Niso et al., 2016), Capilla et al. (2022) developed a voxel-by-voxel atlas of natural frequencies in the resting brain. They showed that the sources of beta oscillations were distributed following a posterior-to-anterior gradient (see Figure 1) showing low-beta (~15–18 Hz) being generated mostly by lateral occipito-parietal regions (Superior Occipital, Middle Occipital, Superior Parietal, and Post-Central Gyri), while high beta oscillations were located in motor (~20 Hz, Pre- and Post-Central Gyri) and prefrontal areas (~20–30 Hz, Middle Frontal Gyrus), corroborating previous findings by Ferrarelli et al. (2012). A complementary approach to unveil region-specific spectral patterns consists in perturbing endogenous oscillations via electrical/magnetic stimulation and recording the EEG response. Transcranial magnetic stimulation (TMS) can be used to transiently increase cortical excitability over specific cortical areas, enhancing their spontaneous oscillatory activity. Samaha et al. (2017) employed this approach and showed that when TMS was delivered to occipital areas, pre-stimulation alpha power predicted TMS-induced phosphenes perception; contrarily, when TMS was delivered to the posterior parietal cortex (PPC), it was the prestimulation beta power to be predictive of phosphenes perception.

### Beta oscillations along the frontoparietal network control spatial attention and eye movements

The M-D stream has direct projections also from early visual cortices to prefrontal cortices (PFC) in primates (Goldman-Rakic and Porrino, 1985; Rempel-Clower and Barbas, 2000) and to inferior frontal gyrus (IFG) (Miller and Cohen, 2001). In addition, the M-D stream has a direct natural continuation in the frontoparietal “dorsal” network for visual attention (Corbetta, 1998; Corbetta and Shulman, 2002; Giesbrecht et al., 2003; Siegel et al., 2008; Shulman et al., 2009; Szczepanski et al., 2010). Bar et al. (2001) suggested that low-level coarse visual representations are rapidly projected from early visual areas to PFC, which then conveys an initial guess of the image back to the temporal cortex, integrating it in the bottom-up flow. Recently, Soyuhos and Baldauf (2023) used resting-state magnetoencephalography (MEG) recordings to directly test the hypothesis that the frontal eyes field (FEF) areas, which is part of the dorsal attention network, has predominant functional coupling with spatiotopically organized regions in the dorsal (“where”) visual stream. They analyzed functional connectivity of spontaneous brain activity, and their results show that FEF has a robust power correlation with the dorsal visual pathway in both the beta and gamma bands. Similarly, beta power modulations in FEF have been associated with frontoparietal feedback activity leading to the suppression of eye movements and attentional shifts, again suggesting an important role of beta band activity in the control of visuospatial attention (Gregoriou et al., 2012; Fiebelkorn et al., 2018; Fiebelkorn and Kastner, 2020).

Modulations of beta oscillations in the fronto-parietal network occur in relation to different visual attention tasks. In particular, beta-band global power at resting state showed correlations with fronto-parietal connectivity and behavioral performance at visual search and gun shooting tasks (Rogala et al., 2020). In addition, early-occurring beta-band connectivity between frontal and parietal regions associated with visual discrimination is suppressed in neglect patients during visual full-field attention tasks (Yordanova et al., 2017).

The predominance of beta functional connectivity along the dorsal visual pathway highlights the relevance of beta oscillations for visual perception, considering also the role of the fronto-parietal network along the M-D stream in the generation of saccadic eye movements. Indeed, saccades depend on the activity of the lateral intraparietal cortex (LIP) in the PPC, responsible for directing spatial attention, and the FEF, responsible for sending motor signal to the superior colliculus, a visuomotor integrative relay innervating the brainstem (Paré and Wurtz, 2001; Curtis and Connolly, 2008). In this network, beta-band oscillations were shown to be fundamental both for saccades preparation and execution, reaching behavior and eye-hand coordination (Dean et al., 2012; Hagan et al., 2012; Mooshagian et al., 2021).

The aforementioned studies frame beta-oscillations within the M-D stream as the core brain rhythm subserving action-oriented behavior, from the orientation of spatial attention, to reaching a target object with gaze and limbs. To this regard, it is important to mention that beta oscillations have been associated with a general mechanism for the maintenance of a motor and/or cognitive state (Engel and Fries, 2010). A possible link between beta oscillations in the dorsal stream and all the key components of action-oriented behaviors may fundamentally lie in the correct perception and maintenance of the representation of space, which is particularly challenging in conditions in which acting in the environment requires a continuous update of such representations. This latter point will be discussed in more detail later (see the *Reconciling old and new perspectives for beta oscillations in visual perception* section).

### Oscillations in the beta band and visuo-spatial perception: correlational and causal evidence

Mounting evidence highlighted a relationship between beta-band activity and perceptual phenomena that require different levels of spatial analysis. Indeed, modulations of beta-band oscillations have been associated with perceptual reorganization (Belitski et al., 2008) such as the one occurring during perceptual switches in bi-stable pictures (Okazaki et al., 2008; Ehm et al., 2011; Kornmeier and Bach, 2012) or even during form-motion integration (Aissani et al., 2014). Of particular relevance, Zaretskaya and Bartels (2015) provided evidence for beta power modulation in PPC during a perceptual switch between global and local motion (and *vice-versa*) in bistable animations, suggesting that beta oscillations reflect the state of individual perceptual set (Engel and Fries, 2010) in terms of visuo-spatial attention and/or feature grouping (Grassi et al., 2018) in moving displays (see Figure 1). In line with other studies using bistable visual stimuli, beta power modulations in parieto-occital sites is found in correspondence of a switch between local and global vision (VanRullen et al., 2006; Yokota et al., 2014; Kloosterman et al., 2015) suggesting that beta oscillations mediate fast large-scale coordination along the dorsal visual stream (Devia et al., 2022).

Further, in a visual crowding task employing letter stimuli, Ronconi et al. (2016) reported a larger post-stimulus beta power reduction in a strong crowding condition (small target-flankers distance) with respect to a weak crowding condition (larger target-flankers distance). In addition, Ronconi and Bellacosa Marotti (2017) showed that the correct performance in the same task was linked to a stronger pre-stimulus beta power (see Figure 1). Ronconi et al. (2020) replicated evidence of an association between letter crowding and beta oscillations in children, by showing an event-related desynchronization (i.e., power reduction) in the beta band spanning both pre- and post- stimulus time windows. This beta power reduction was not evident in a group of children with autism spectrum disorders, potentially suggesting an impaired frontoparietal top-down influence on ventral stream regions as a signature of their hyperlocal perception.

The aforementioned studies provide important correlational evidence about the role of beta oscillations in visuo-spatial perception. However, casual links should be inferred when behavioral changes are observed following a direct modulation of neural oscillations. Transcranial Alternating Current Stimulation (tACS) represents one promising medium to achieve this purpose for its capacity to modulate the activity of specific frequency bands via neuronal entrainment (Helfrich et al., 2014; Witkowski et al., 2016; Lakatos et al., 2019). Battaglini et al. (2020) showed that right parietal beta-band (18 Hz) tACS, as compared to alpha tACS (10 Hz) and sham (placebo) stimulation ameliorated performance in a visual crowding task when crowded displays were presented to the contralateral hemifield. This study provided the first causal evidence for the involvement of beta oscillations in visual crowding corroborating the link between beta activity and visuo-spatial perception.

The relevance of beta-band oscillations in fronto-parietal regions during visual perception is further corroborated by multiple transcranial magnetic stimulation (TMS) studies. Modulating beta oscillations via repetitive TMS (rTMS) in right parietal cortex facilitated local processing in a Navon task (Romei et al., 2011; see Figure 1), in accordance with the global–local switch in bi-stable motion shown by Zaretskaya and Bartels (2015). More recently, it has been shown that single-pulse TMS applied to frontal eye fields (FEF) triggers phase reset of beta oscillations at occipital sensors and modulate the accuracy of motion discrimination (Veniero et al., 2021), indicating that the activity of frontal lobes exerts a top-down influence on parietal activity shaping visual perception. Relatedly, rTMS at high beta frequency (30 Hz) applied to FEF resulted in higher inter-regional synchronization in beta oscillations between FEF and bilateral parietal sensors and increased visual sensitivity in a visual detection task (Stengel et al., 2021), while low beta rTMS (20 Hz) delivered to right Intraparietal sulcus (IPS) or right FEF interfered with visual identification (Capotosto et al., 2009). Thus, top-down beta-band rhythmic communication within the fronto-parietal network appears as having an evident role for visual perception and attention, in agreement with its putative role for the dorsal stream functionality.

### The role of beta oscillations in reading, developmental dyslexia, and related visuo-attentional impairments

The functions of the M-D stream are crucial for reading and its acquisition (Vidyasagar and Pammer, 2010; Gori et al., 2016; Stein, 2019). In this context, the parietal cortex facilitates attention-demanding letters/words identification grounded in the ventral stream by providing coarse spatial representations allowing for the segregation of target items from the other neighboring ones. In this way, speech sounds can be finally mapped to single letters, syllables and words. To this regard, the magnocellular theory of developmental dyslexia (Stein and Walsh, 1997; Stein, 2019) suggests that alterations of the M-D pathway have a primary role in determining reading impairments but also impaired visual search (Vidyasagar, 1999) and visuo-spatial attention (Visser et al., 2004; Vidyasagar and Pammer, 2010). A few studies reported alterations in beta oscillations as well as in other frequency bands in individuals with DD during linguistic tasks tapping into phonological, orthographic and semantic processing (Rippon, 2000; Klimesch et al., 2001; Spironelli et al., 2008), but also in auditory rhythmic tracking tasks (De Vos et al., 2017; Chang et al., 2021). Importantly, Turri et al. (2023) reported lower resting-state beta power over parieto-occipital sites in adults with DD, and showed that beta power in these cortical sites was a predictor of reading accuracy in DD but not in typical readers.

Collectively, these findings suggest that the study of beta oscillations’ functional role in the dorsal stream might foster the development of new treatment approaches to visuo-attentional and reading impairment, possibly aiming at modulations of beta-band activity.

### Reconciling old and new perspectives for beta oscillations in visual perception

In their seminal work, Engel and Fries (2010) outlined the first theoretical framework describing the role of beta oscillations for motor and perceptual functions. In their view, beta oscillations reflect the maintenance of the “status-quo” of a motor set while preventing the execution of new movements and favouring the re-establishment of such sets post-movement. They further extend this proposal to the perceptual domain suggesting that beta oscillations act in the same ways in tasks requiring top-down control, which can be perturbed when behavioural responses depend mainly on exogenous factors. This particular view possibly depicts beta oscillations as a sustained rhythm acting in a putatively passive/resistive way. In a following work, Fries (2015) further suggested that beta oscillations may have a rather active role in feedback loops modulating gamma band activity and interareal communication through synchronization. Notably, the notion of beta oscillations as a continuous rhythm has been challenged by recent works depicting beta as emerging in brief transient bursts (<150 ms) in frontal (Lundqvist et al., 2016), somatosensory (Jones et al., 2009; Ziegler et al., 2010; Sherman et al., 2016), motor (Feingold et al., 2015; Rule et al., 2017), and occipital (Freyer et al., 2009) areas, across species and recording modalities (Sherman et al., 2016; Shin et al., 2017; Bonaiuto et al., 2021; Rassi et al., 2023a,b).

Capitalizing on the role of beta oscillations as a rather “active” rhythm underlying interareal communication (Donner and Siegel, 2011; Fries, 2015; Jensen et al., 2015; Sherman et al., 2016; Palmigiano et al., 2017; Palva and Palva, 2018) and top-down processing (Shulman et al., 2009; Bastos et al., 2015), which would appear in transient bursts across different cortical sites, Spitzer and Haegens (2017) developed a new theoretical framework inspired by several works showing the involvement of beta oscillations in working memory and decision making within the somatosensory domain. Specifically, beta oscillations were associated with the maintenance, reactivation and updating of content-specific representations of scalar magnitudes pertaining to different stimulus features in the vibrotactile, auditory and visual domains (Spitzer et al., 2010, 2014; Rose et al., 2016; Vergara et al., 2016; Wimmer et al., 2016; Shin et al., 2017; Rassi et al., 2023a,b). In Spitzer and Haegens’ perspective, considering the association between beta oscillations and top-down processing (Shulman et al., 2009; Bastos et al., 2015), their suitability to govern long-range communication (Donner and Siegel, 2011; Fries, 2015; Jensen et al., 2015; Sherman et al., 2016; Palmigiano et al., 2017; Palva and Palva, 2018), and their putative role in the formation of cell ensembles and their firing patterns (Roopun et al., 2008; Kopell et al., 2011), beta oscillations essentially support the endogenous re-activation of cortical representations acting as a short-lived mediator within and between cortical ensembles.

Such transient (burst-like) nature of beta oscillations offers the possibility to discuss previous studies linking beta oscillations and visual perception in light of less and more recent theoretical accounts. Perceptual sets (e.g., as in Engel and Fries, 2010) could putatively be ascribable to a set of high-level information concerning the organization of perceptual input, hence encodable in a memory trace that may be active or latent. Several works reviewed in the present work reported correlational (VanRullen et al., 2006; Yokota et al., 2014; Zaretskaya and Bartels, 2015) and causal (Capotosto et al., 2009; Romei et al., 2011; Battaglini et al., 2020; Veniero et al., 2021) involvement of frontoparietal/parietal beta oscillations in visual tasks in which spatial information must be maintained in working memory and routed to other networks to exert top-down influence, rapidly updated in the form of a new perceptual set. While the evidence about the transient nature of beta oscillations in the parietal cortex and/or the visual pathways is scarce, it could in principle support the need of the visual system to actively monitor and update spatial information and rapidly route it across the visual streams.

## Conclusion

Historically, alpha oscillations have been the most well characterized brain rhythm, and also the most studied in the context of visual perception. In the present review, we have summarized the emerging evidence showing a preliminary, but at the same time coherent, picture of the role of beta oscillations in the context of visual perception. We have shown that beta oscillations are modulated in a wide range of visual tasks that require a precise spatial representation of visual input, specifically where forming a faithful representation of its spatial coordinates is essential. These include tasks where visual elements need to be perceptually reorganized, such as in the case of bistable picture, when there is a need to switch from a global to a local level of visual analysis (and vice versa), when there is a need to spatially segregate stimuli in cluttered visual scenes, such as in the case of crowding, and finally, when there is a need to extrapolate visual motion and accomplish form-motion integration. All these are conditions where beta oscillations have been shown to play a crucial role, supporting the claim that this brain rhythm is primarily serving the activity of the magnocellular-dorsal (or “where”) visual pathway. The role of beta oscillations can be seen as complementary to the function of alpha, which would be the preferential rhythm supporting the combination of object features into longer neurocomputational cycles to merge emerging representations from distributed areas within the ventral (or “what”) visual pathway.

Framing beta-oscillations as the main rhythm of the dorsal stream has of course also implications for action-oriented behaviors (perception for action), from the orientation of spatial attention, to eyes and body movement. The proposal of beta oscillation as the rhythm of the dorsal stream reconciles the emerging evidence that beta-band rhythmic communication has a key role within the fronto-parietal dorsal attentional network. It remains an open question (Table 1) whether it is possible to differentiate a ‘perceptual’ and an ‘attentional’ beta rhythm, or whether they are orchestrated in partially different neural networks, but with a common underlying rhythmic neural code. Regarding eye and upper limb movements, increasing evidence show that beta-band oscillations in both parietal (PPC) and frontal (FEF) areas are fundamental both for saccades execution, reaching behavior and eye-hand coordination, reconciling our perspective with the idea that beta oscillations are important for the maintenance of a particular motor and/or cognitive state (Engel and Fries, 2010). A key underlying component of different action-oriented behaviors may be, indeed, the setting, maintaining and continuously updating of a faithful representation of spatial coordinates. In this sense, fast updating of spatial information could exploit the burst-like properties of beta oscillations, which according to mounting evidence is a key distinctive feature that differentiate beta from alpha oscillations in different sensory modalities.

**Table 1:** Open questions regarding beta oscillations in the magnocellular dorsal visual pathway.

Open questions
Perceptual segregation/integration and visuo-spatial attention: unique or differentiated functions of beta oscillations? What is the relationship between alpha and beta-band oscillations considering the existence of ‘bimodal’ areas in which alpha and beta bands are both predominant frequencies? Is it possible to use neuromodulation to treat clinical conditions characterized by reading, visuo-spatial (e.g., impaired objects segregation) and visuo-attentional impairments? Is it possible to extend the role of beta band activity found in visual perception to other sensory modalities and multisensory processing? Do beta oscillations have a role in mediating the formation and reactivation of memory traces related to visuo-spatial processes? Can perceptual sets be ascribed to memory traces?

Although there are no studies to date designed to address the relationship between beta oscillations and perceptual awareness, a reasonable speculation is that such dorsal beta activity in vision acts at a preconscious level. This seems plausible considering that it is a fast rhythm that would convey only a transient, coarse and undetailed representation of the visual scene and its spatial properties toward a more distributed network, fostering attention-demanding object identification in ventral stream areas at the speed of the (slower) alpha rhythm.

Finally, the growing literature on the role of beta oscillation in visual perception in healthy individuals is corroborated also by initial studies highlighting anomalies of beta oscillations in developmental dyslexia, a neurodevelopmental condition where the core visuo-attentional deficits are widely recognized to arise from a M-D stream deficit. Thus, targeting beta oscillations in the dorsal visual stream seems a promising new rehabilitative strategy, especially considering the different neuromodulatory (tACS/TMS) studies that have effectively modulated beta oscillations with functional consequences for perception in the typical population.

## Author contributions

GD: Conceptualization, Writing – original draft, Writing – review & editing, Visualization. LR: Conceptualization, Writing – original draft, Writing – review & editing, Funding acquisition, Supervision.
